# Supplementary material for: Fibroblast Growth Factor 21 as a Potential Biomarker for Improved Locomotion and Olfaction Detection Ability after Weight Reduction in Obese Mice
Source: Nutrients. 2021 Aug 24;13(9):2916. doi: 10.3390/nu13092916 (PMC8470262; doi:10.3390/nu13092916)
Supplement: Supplementary file 1 [file nutrients-13-02916-s001.zip › Supplement_S1_S2_S5.pdf]

Supplementary Tables

S1 - Table: Experimental parameters used for PCA, Multidimensional Heatmap and ML feature selection. Parameters were aligned to a numeric number.

**N°**	**0**	**1**	**2**	**3**	**4**
**Data**	FGF21 conc. [pg/mL]	body weight [g]	visceral fat [g]	subcutaneous flanked. fat [g]	fasting time [min]
**Experiment**	FGF21	body comp.	body comp.	body comp.	BPT
**N°**	**5**	**6**	**7**	**8**	**9**
**Data**	lick/eat pellet [yes/no]	latency to lick/eat pellet [sec]	fasting time [min]	lick/eat pellet [yes/no]	latency to lick/eat pellet [sec]
**Experiment**	BPT	BPT	BPT/SPT	BPT/SPT	BPT/SPT
**N°**	**10**	**11**	**12**	**13**	**14**
**Data**	cum. duration open arm [%]	cum. duration closed arm [%]	cum. duration centre [%]	total entries [counts]	entries open arm [%]
**Experiment**	EPM	EPM	EPM	EPM	EPM
**N°**	**15**	**16**	**17**	**18**	**19**
**Data**	entries closed arm [%]	entries centre [%]	vertical activity [counts]	mean velocity [cm/s]	distance moved [cm]
**Experiment**	EPM	EPM	EPM	EPM	EPM
**N°**	**20**	**21**	**22**	**23**	**24**
**Data**	Moving time [%]	Immobility [%]	cum. duration centre [%]	cum. duration outer zone [%]	total entries [counts]
**Experiment**	EPM	EPM	OF	OF	OF
**N°**	**25**	**26**	**27**	**28**	**29**
**Data**	entries centre [%]	entries outer zone [%]	vertical activity [counts]	mean velocity [cm/s]	distance moved [cm]
**Experiment**	OF	OF	OF	OF	OF
**N°**	**30**	**31**			
**Data**	Moving time [%]	Immobility [%]			
**Experiment**	OF	OF			

S2 - Table: Classification report of ML algorithms

**whole data set**					
ML algorithm	precision weighted average	recall weighted average	F1-Score weighted average	6 fold CV (of recall)	Best Params
Logistic Regression*	0.61	**0.65**	0.57	**0.63**	C': 0.01, 'penalty': 'l2', 'solver': 'liblinear'
SGD Classifier	0.61	**0.65**	0.59	**0.58**	SGDClassifier()
Neural Network*	0.53	**0.59**	0.53	**0.61**	activation': 'logistic', 'hidden_layer_sizes': 20, 'learning_rate_init': 0.005, 'max_iter': 300, 'solver': 'sgd'
SVC Classifier*	0.53	**0.59**	0.53	**0.59**	'C': 1, 'gamma': 'scale', 'kernel': 'rbf'
Random Forest*	0.50	**0.53**	0.47	**0.59**	'criterion': 'entropy', 'max_depth': 4, 'max_features': 'log2', 'n_estimators': 100
Decision Tree*	0.50	**0.53**	0.47	**0.53**	'criterion': 'gini', 'max_leaf_nodes': 6, 'min_samples_split': 2
Naive Bayes	0.53	**0.53**	0.47	**0.53**	GaussianNB()
Gradient Boosting	0.54	**0.41**	0.43	**0.41**	n_estimators=20, learning_rate=1.0, max_depth=3, random_state=0
**Feature Selected (FS) data**					
ML algorithm	precision weighted average	recall weighted average	F1-Score weighted average	6 fold CV (of recall)	Best Params
Neural Network*	0.82	**0.76**	0.78	**0.72**	activation': 'identity', 'hidden_layer_sizes': 90, 'learning_rate_init': 0.001, 'max_iter': 200, 'solver': 'sgd'
Logistic Regression*	0.71	**0.71**	0.71	**0.71**	C':1, 'penalty':'l1', 'solver':'saga'
Gradient Boosting*	0.78	**0.71**	0.72	**0.71**	criterion': 'friedman_mse', 'learning_rate': 0.05, 'max_depth': 6, 'max_features': 'sqrt', 'n_estimators': 30
Naive Bayes	0.69	**0.71**	0.69	**0.70**	GaussianNB()
Random Forest	0.76	**0.71**	0.72	**0.70**	n_estimators= 20, max_depth=3, random_state=0
SVC Classifier*	0.66	**0.65**	0.65	**0.65**	C':0.1, 'gamma':'scale', 'kernel':'linear'
Decision Tree	0.73	**0.65**	0.65	**0.64**	class_weight=None, max_depth=4, max_features=None, max_leaf_nodes=None, min_impurity_decrease=0.0, min_impurity_split=None, min_samples_leaf=1, min_samples_split=2, min_weight_fraction_leaf=0.0, presort=False, random_state=42, splitter='best'
SGD Classifier*	0.68	**0.65**	0.65	**0.62**	alpha': 0.001, 'max_iter': 100, 'n_jobs': -1, 'penalty': 'elasticnet'

** = Hyperparameter Tuned*

S3 - Table: Correlation matrix of multidimensional heatmap with correlation values

see excel sheet S2_table_correlation_matrix

S4 - Table: PCA values

see excel sheet S3_table_PCA_values

S5 - Table: Selected features

Chi2	Ridge	RFE
FGF21 (FGF21)	FGF21 (FGF21)	FGF21 (FGF21)
body weight (body comp.)	body weight (body comp.)	body weight (body comp.)
visceral fat (body comp.)	visceral fat (body comp.)	visceral fat (body comp.)
sub. flanked fat (body comp.)	sub. flanked fat (body comp.)	sub. flanked fat (body comp.)
lick/eat pellet (BPT)	fasting time (BPT)	fasting time (BPT)
latency to lick/eat pellet (BPT)	latency to lick/eat pellet (BPT)	latency to lick/eat pellet (BPT)
lick/eat pellet (SPT)	fasting time (SPT)	fasting time (SPT)
latency to lick/eat pellet (SPT)	latency to lick/eat pellet (SPT)	latency to lick/eat pellet (SPT)
cum. duration, open arm (EPM)	cum. duration, closed arm (EPM)	vertical activity (OF)
distance moved (EPM)	vertical activity (EPM)	moving time (OF)
total entries (OF)	vertical activity (OF)	
vertical activity (OF)	mean velocity (OF)	
mean velocity (OF)	distance moved (OF)	
distance moved (OF)	moving time (OF)	
moving time (OF)		
**Total: 15**	**Total: 14**	**Total: 10**
